# Green People

**Published:** 1860-01

**Authors:** 


### GREEN PEOPLE.

SOME people are as green as the grass they tread upon; or, to change the simile, their noggins are as soft as mush; it is a wonder they have sense enough to breathe. An individual in this city has an amount of stupidity which is perfectly refreshing, that is, to one who possesses a temperament like our own, which breaks out into a horse laugh as loud as a fourth of July torpedo whenever any thing turns up, which is too insignificant to be ruffled at, but is so unmistakably simple, as to put one in a betweenity whether to laugh or growl; and as no man of the least common-sense would choose the latter when he could just as well indulge in a loud guffaw, so we did have a little quiet merry-making in receiving a note from the penny-post, charge two cents, from a name we never heard of, to the effect that the writer from reading a piece of ours headed Long Life, would be greatly obliged for our opinion as to whether "smoking" was injurious to health, and to send a note, with the answer, stating at the same time whether tobacco was a poison. All we can say is, that we do not think that any thing could "poison" this gentleman—we think he must be "impervious" to the effects of any toxological agent known to us or to any one else. We really do not think he is capable of being hurt at all. While we are among the stupidities, brief mention may be made of one of constant occurrence, and which, by the way, puts a good many dollars into our pocket in the way of editorial perquisites. We receive from one to five or ten or more dollars at a time from persons whom we do not know, in the way of subscriptions, purchases of books, opinions, medical advice, etc.; but the obstacle to our compliance is in the little item, that the name of the person is not mentioned, or the post-office address is omitted altogether as to State, county, or town.

